# Seasonal variations for newly prescribed urate-lowering drugs for asymptomatic hyperuricemia and gout in Japan

**DOI:** 10.3389/fphar.2024.1230562

**Published:** 2024-01-16

**Authors:** Masafumi Kurajoh, Seigo Akari, Takashi Nakamura, Yasutaka Ihara, Takumi Imai, Tomoaki Morioka, Masanori Emoto

**Affiliations:** ^1^ Department of Metabolism, Endocrinology and Molecular Medicine, Osaka Metropolitan University Graduate School of Medicine, Osaka, Japan; ^2^ Medical Affairs Department, Sanwa Kagaku Kenkyusho Co., Ltd., Nagoya, Aichi, Japan; ^3^ Department of Medical Statistics, Osaka Metropolitan University Graduate School of Medicine, Osaka, Japan

**Keywords:** seasonality, prescription, asymptomatic hyperuricemia, gout, urate-lowering drugs

## Abstract

**Background:** Urate-lowering drugs (ULDs) have been approved for treatment of asymptomatic hyperuricemia and gout in Japan. Although serum urate levels and rates of gout onset are known to have seasonal variations, no survey results regarding the seasonality of ULD prescriptions for asymptomatic hyperuricemia and gout have been reported.

**Methods:** A large-scale database of medical claims in Japan filed between January 2019 and December 2022 was accessed. In addition to total size of the recorded population for each month examined, the numbers of patients every month with newly prescribed ULDs for asymptomatic hyperuricemia and gout were noted, based on the International Classification of Diseases, 10th Revision, codes E79.0 and M10.

**Results:** The results identified 201,008 patients with newly prescribed ULDs (median age 49.0 years, male 95.6%). Of those, 64.0% were prescribed ULDs for asymptomatic hyperuricemia and 36.0% for gout. The proportion of new ULD prescriptions was seasonal, with that significantly (*p* < 0.001) higher in summer (June–August) [risk ratio (RR) 1.322, 95% CI 1.218 to 1.436] and autumn (September–November) (RR 1.227, 95% CI 1.129–1.335) than in winter (December–February), whereas the proportion in spring (March–May) was not significantly different from winter. There was no significant difference after stratification by drug type (uric acid production inhibitor/uricosuric agent) or size of the medical institution, nor subgrouping by age or sex (p for interaction = 0.739, 0.727, 0.886, and 0.978, respectively). On the other hand, the proportions of new ULD prescriptions for asymptomatic hyperuricemia were significantly lower and for gout significantly higher in spring than winter, while those were similar in summer and autumn for both groups (p for interaction<0.001).

**Conclusion:** The present findings indicate that new prescriptions for ULDs to treat asymptomatic hyperuricemia or gout in Japan show seasonal differences, with higher rates noted in summer and autumn as compared to winter.

## Introduction

The rates of prevalence of asymptomatic hyperuricemia and gout, caused by prolonged hyperuricemia, are increasing throughout the world (Putterman, 1991; Hakoda, 2012; Kuo et al., 2015; Elfishawi et al., 2018; Hakoda and Kasagi, 2019; She et al., 2022). Based on accumulating evidence, the Japanese Guidelines for Management of Hyperuricemia and Gout, 3^rd^ edition, recommend administration of urate-lowering drugs (ULDs) for patients with asymptomatic hyperuricemia complicated by renal disease as well as gout patients in addition to lifestyle modification (Hisatome I et al., 2020). Allopurinol, febuxostat, topiroxostat, benzbromarone, probenecid, and dotinurad have been approved and are used as ULDs in Japan.

Variations in serum urate levels and gout attacks related to season of the year have been reported, with most studies showing higher urate levels and a greater proportion of gout attacks in summer and autumn (Elliot et al., 2009; Karmacharya et al., 2016; Akerblom et al., 2017; Romaszko et al., 2022). Those findings suggest that the frequency and degree of hyperuricemia are higher in summer and autumn, thus the frequency of gout attacks is consequently also higher in summer and autumn. However, no real-world data have been presented regarding whether ULD prescriptions in daily clinical practice for asymptomatic hyperuricemia and gout also have seasonal variations.

Using a large medical claims database of cases in Japan, the present study was conducted to investigate seasonal variations in prescriptions of ULDs for treatment of asymptomatic hyperuricemia and gout by determining the numbers of monthly prescriptions of new ULDs for asymptomatic hyperuricemia and gout.

## Subjects and methods

### Study design and data source

This was an observational study that used the JMDC Claims Database (JMDC Inc., Tokyo, Japan) (Kimura et al., 2010), which as of December 2022 contained data for over 9.8 million individuals. Information from various health insurance societies related to individuals aged ≤74 years in Japan with health insurance, including employed people and their families, is stored in the database, and includes prescription details, such as name of drug, number of days, and prescribing source, as well as patient information, such as age, sex, and International Classification of Diseases 10th Revision (ICD-10) diagnosis, and also procedure codes. For this study, anonymized information obtained from the commercially available JMDC Claims Database was used in accordance with the Japan Act on the Protection of Personal Information, with individual informed consent not required for its provision or use. According to the ethical guidelines for clinical research in Japan, studies using such anonymized processed information do not require a review by an ethics review committee. The present study was conducted in full accordance with the principles of the Declaration of Helsinki.

### Data extraction

**FIGURE 1:**
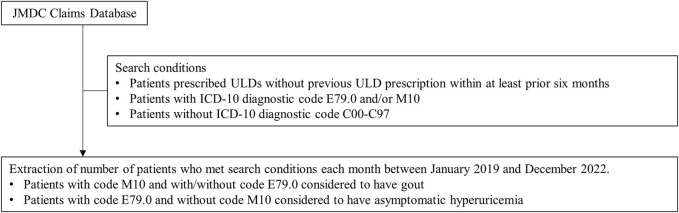
Search conditions for use of JMDC Claims Database. ULDs: allopurinol, febuxostat, topiroxostat, benzbromarone, probenecid, dotinurad Abbreviations: ULDs, urate-lowering drugs; ICD-10, International Classification of Diseases 10th Revision.

The contract for the commercially available JMDC data used in the present study allowed for numbers of patients to be extracted according to age, sex, ICD-10 diagnosis code, and prescription details, including drug name and prescribing source, from 6 months to 5 years prior to the date of search enforcement. In addition to noting the total population number for each month, the numbers of patients newly prescribed ULDs for asymptomatic hyperuricemia and gout were extracted from the database for every month between January 2019 and December 2022. Search conditions were individuals (1) prescribed ULDs (allopurinol, febuxostat, topiroxostat, benzbromarone, probenecid, dotinurad) who did not have a previous ULD prescription within at least the prior 6 months, (2) with an ICD-10 diagnostic code for hyperuricemia without signs of inflammatory arthritis and tophaceous disease (E79.0) and/or gout (M10), and (3) without an ICD-10 diagnostic code for malignant neoplasms (C00-C97) (Figure 1). The same data extraction procedures were repeated after stratifying the entire population by age and sex. In addition, clinical characteristics of patients with a new ULD prescription and related details, such as disease type (asymptomatic hyperuricemia/gout), drug type (uric acid production inhibitor/uricosuric agent), and size of the medical institution issuing the prescription, were also obtained.

### Definition of asymptomatic hyperuricemia and gout

Gout is caused by a condition known as hyperuricemia, thus patients with a code for gout (M10) with or without a code for hyperuricemia (E79.0) were considered to have gout. On the other hand, those with a code for hyperuricemia (E79.0) without a code for gout (M10) were considered to have asymptomatic hyperuricemia (Figure 1).

### Uric acid production inhibitors and uricosuric agents

Among the prescribed ULDs, allopurinol, febuxostat, and topiroxostat were classified as uric acid production inhibitors, while benzbromarone, probenecid, and dotinurad were classified as uricosuric agents (Hisatome I et al., 2020; Hosoya et al., 2020).

### Classification of seasons

Following the classification method of the Japan Meteorological Agency, the year was divided into four seasons; Spring (March-May), Summer (June-August), Autumn (September-November), and Winter (December-February).

### Other clinical assessments

Medical institutions with more than 100 beds were categorized as large and medium hospitals, while those with less than 100 beds were categorized as small hospitals and clinics, as previously described (Wada et al., 2013). The presence of hypertension, diabetes, dyslipidemia, renal failure, cardiovascular disease, heart failure, and cerebrovascular disease was identified by the ICD-10 diagnostic codes I10-15, E10-14, E78, N17-19, I20-25, I50, and I60-69, respectively. In addition, administration of drugs known or suspected to affect serum uric acid levels, such as losartan, fenofibrate, sodium-glucose cotransporter 2 (SGLT2) inhibitors, diuretics, beta-blockers, and nonsteroidal anti-inflammatory drugs (NSAIDs), was also assessed.

### Statistical analysis

Demographics and clinical characteristics of patients with a new ULD prescription are expressed as numbers (percentages) for categorical variables and median values (interquartile range) for continuous variables. The proportion of patients with a new ULD prescription for each month was calculated by dividing the number of patients with a new ULD prescription each month by the total size of the population in the JMDC database each month. The number with new a ULD prescription was then analyzed using Poisson regression with dispersion parameter, using total size of population (log scale) as the offset, seasonality as the explanatory variable, and year as the adjustment variable. The presence or absence of seasonality in the proportion of patients with a new ULD prescription was examined using an analysis of variance (ANOVA) method, with risk ratios (RRs) for a new prescription in spring, summer, and autumn compared with winter. These analyses were repeated following further classification of new ULD prescription by disease type (asymptomatic hyperuricemia/gout), drug type (uric acid production inhibitors/uricosuric agents), and size of the medical institution that issued the prescription (large and medium-sized hospitals/small hospitals and clinics). Subgroup analyses were also performed according to age (0–39, 40–59, 60–74 years) and sex (male/female).

The R software package, version 3.6.3 (R Foundation for Statistical Computing, Vienna, Austria), was used for data analysis. All reported *p* values are two-tailed and were considered statistically significant at <0.05.

## Results

### Subjects

There were 201,008 patients with newly prescribed ULDs for asymptomatic hyperuricemia and gout identified in the database during the study period.

### Clinical characteristics

Characteristics of the enrolled subjects are shown in Table 1. The median age was 49.0 years and 95.6% were male. Of the ULD prescriptions, 64.0% were given for asymptomatic hyperuricemia and 36.0% for gout, while type of ULD prescribed was uric acid production inhibitor in 91.2% and uricosuric agent in 9.5%. Large and medium-sized hospitals were the source of 16.3% of the ULD prescriptions, while small hospitals and clinics were the source of 83.6%. The rates of prevalence for hypertension, diabetes mellitus, dyslipidemia, renal failure, cardiovascular disease, heart failure, and cerebrovascular disease was 34.8%, 15.9%, 41.7%, 3.3%, 3.5%, 4.3%, and 2.9%, respectively, while the rates of prescription for losartan, fenofibrate, SGLT2 inhibitors, diuretics, beta-blockers, and NSAIDs were 0.7%, 0.4%, 1.5%, 3.0%, 3.5%, and 8.1%, respectively.

**TABLE 1:** Clinical characteristics of patients newly prescribed ULDs (*n* = 201,008).

Age, years	49.0 (41.0–56.0)
Age group, n (%)	
0–39 years	42,178 (21.0)
40–59 years	126,303 (62.8)
60–74 years	32,527 (16.2)
Sex, n (%)	
Male	192,084 (95.6)
Female	8,924 (4.4)
Disease type, n (%)	
Asymptomatic hyperuricemia	128,650 (64.0)
Gout	72,358 (36.0)
ULDs, n (%)	
Uric acid production inhibitors	183,325 (91.2)
Uricosuric agents	19,041 (9.5)
Medical institutions, n (%)	
Large and medium sized hospitals	32,864 (16.3)
Small hospitals and clinics	168,082 (83.6)
Unknown	62 (0.03)
Coexisting conditions, n (%)	
Hypertension	69,936 (34.8)
Diabetes mellitus	31,988 (15.9)
Dyslipidemia	83,819 (41.7)
Renal failure	6,727 (3.3)
Cardiovascular disease	7,083 (3.5)
Heart failure	8,686 (4.3)
Cerebrovascular disease	5,734 (2.9)

Values are expressed as median (interquartile range) for continuous variables or number (percentage) for categorical variables.

Abbreviations: ULDs, uric acid-lowering drugs.

### Seasonality for new ULD prescriptions

**FIGURE 2:**
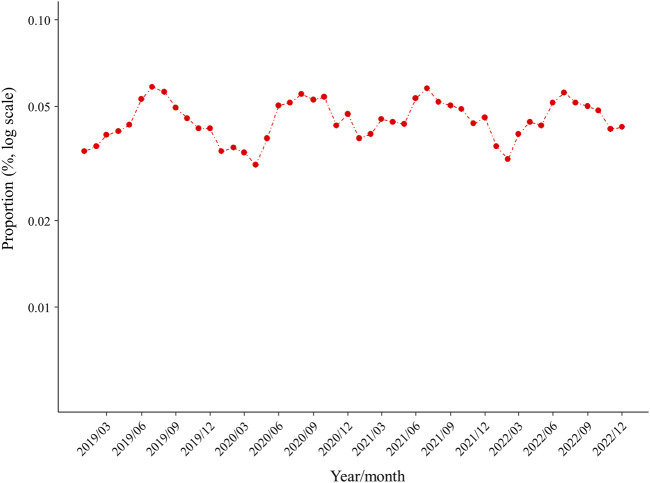
Proportion of prescriptions for new ULDs each month. Abbreviations: ULDs, urate-lowering drugs.

**FIGURE 3:**
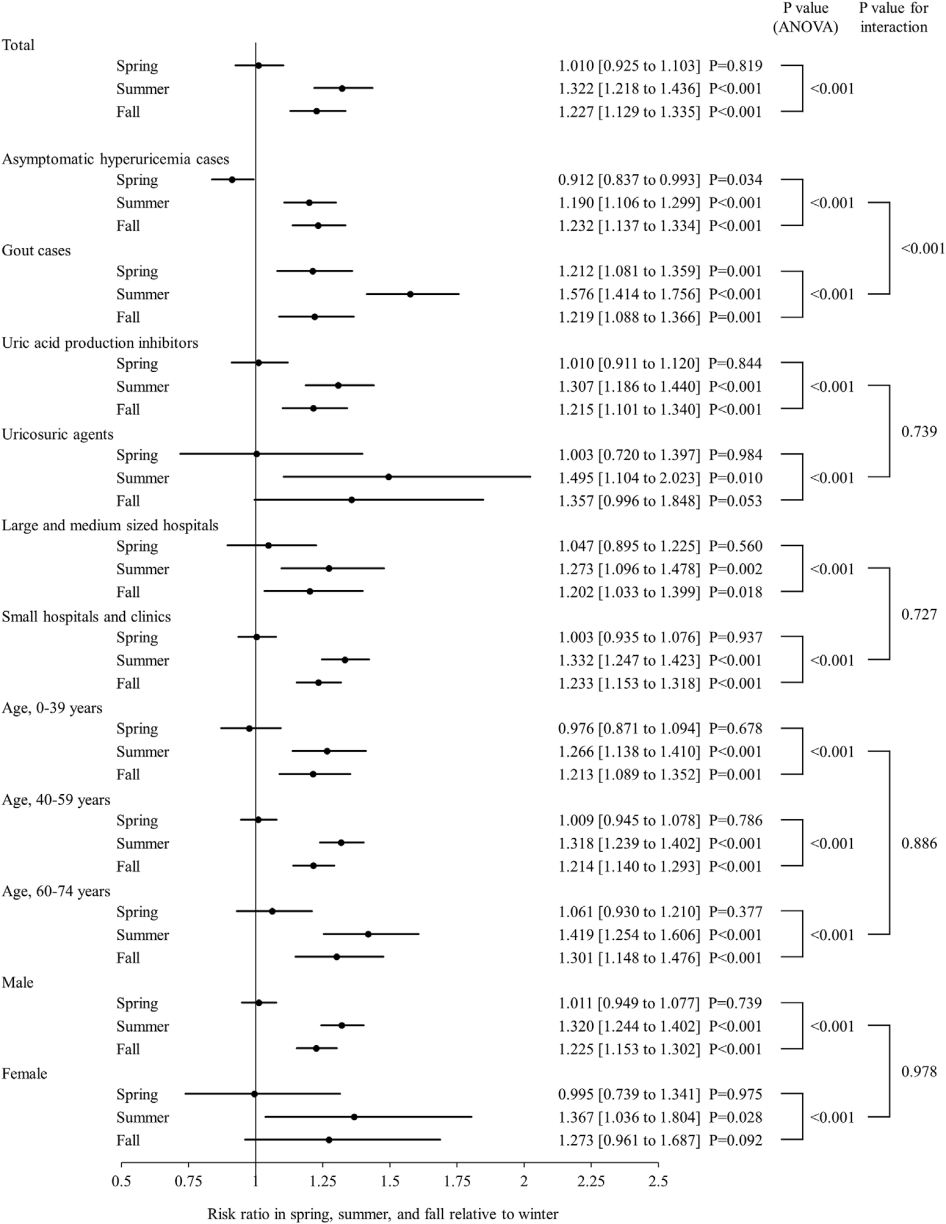
Seasonal analysis of new ULD prescriptions with total, stratified, and subgroup data shown. Forrest plots showing risk ratio and 95% confidence interval as circles and straight lines, respectively, for spring, summer, and autumn relative to winter. Abbreviations: ULDs, urate-lowering drugs; ANOVA, analysis of variance.

**FIGURE 4:**
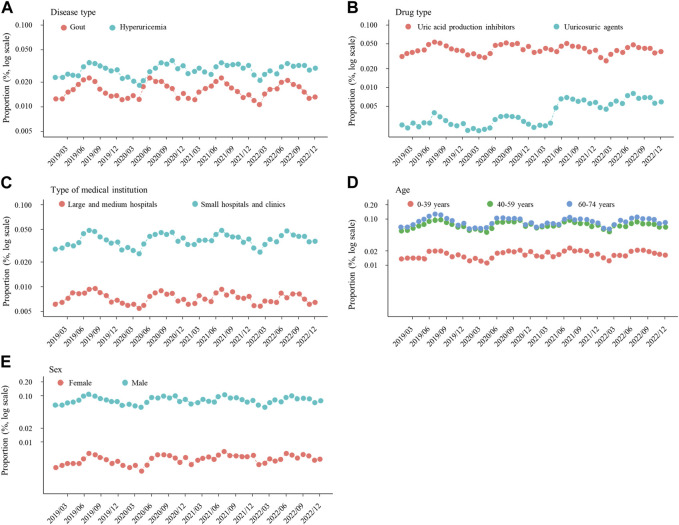
Proportion of prescriptions for new ULDs each month after stratification by **(A)** disease type, **(B)** drug type, and **(C)** medical institution, and also subgrouping by **(D)** age and **(E)** sex. Abbreviations: ULDs, urate-lowering drugs.

Figure 2 shows the proportion of new ULD prescriptions for each month during the study period, which was apparently higher in summer and lower in winter. ANOVA findings showed significant seasonality for ULD prescriptions (*p* < 0.001), with the proportions in summer (RR 1.322, 95% CI 1.218–1.436) and autumn (RR 1.227, 95% CI 1.129–1.335) significantly higher as compared to winter (*p* < 0.001), whereas the proportion of ULD prescriptions in spring was not significantly different from that in winter (Figure 3). When new prescriptions of ULDs were further categorized by drug type and size of the medical institution issuing treatment, a similar seasonality was observed for all types of prescriptions (Figures 3, 4), with no notable heterogeneity among the categories found (p for interaction = 0.739 and 0.727, respectively). Notably, when stratified by asymptomatic hyperuricemia and gout, the proportion of new ULD prescriptions in summer and autumn were significantly higher than in winter in both groups, while the proportion of new ULD prescriptions for asymptomatic hyperuricemia were significantly lower and for gout significantly higher in spring as compared to winter (p for interaction<0.001) (Figure 3).

### Subgroup analyses

There were no notable inconsistencies for the main results observed among the age and sex subgroups (p for interaction = 0.886 and 0.978, respectively), with significant seasonality also observed for those (Figures 3, 4).

## Discussion

This is the first known study to investigate seasonal variations in prescriptions of ULDs for asymptomatic hyperuricemia and gout. The findings showed seasonal variations of new prescriptions for ULDs, as they were significantly more common in summer and autumn as compared winter, with no notable difference after stratification by disease type, drug type, and medical institution, or subgrouping by age and sex (Figures 2–4). On the other hand, as compared to winter, new ULD prescriptions for asymptomatic hyperuricemia were significantly lower and for gout significantly higher in spring.

Previous reports have noted differences regarding proportion and persistence of ULD prescriptions for asymptomatic hyperuricemia and/or gout, as well as type and cost (Hakoda and Kasagi, 2019; Peng et al., 2019; Koto et al., 2021; Akari et al., 2022). However, no real-world data on the seasonality of ULD prescriptions have been presented. The present study indicates that new prescriptions of ULDs for asymptomatic hyperuricemia and gout are seasonal, with higher rates of prevalence in summer and autumn (Figures 2, 3), with no remarkable difference found following stratification of the results based on asymptomatic hyperuricemia and gout (Figures 3, 4). These findings suggest a relationship with seasonal onset of asymptomatic hyperuricemia and gout, as clinicians prescribe ULDs for those conditions more often in summer and autumn.

Seasonality in regard to serum urate levels and onset of gout attacks has been reported. A study of urate levels in more than 170,000 blood samples from a Swedish adult population showed higher levels in those obtained in summer (Akerblom et al., 2017), with other similar findings also presented (Goldstein et al., 1972; Letellier and Desjarlais, 1982; Murciano Revert et al., 2000). In addition, a study that examined serum urate in 54,536 blood specimens from a Polish laboratory showed higher levels in autumn samples (Romaszko et al., 2022). In regard to the seasonality of gout incidence, a large study that analyzed surveillance data for 920,000 patients found that new cases of gout were more frequent in summer (Elliot et al., 2009), which has been confirmed in other similar reports (Goldstein et al., 1972). In addition, a study of 28,172 patients admitted to a hospital with a primary diagnosis of acute gouty arthritis showed that gout onset was more common in autumn (Karmacharya et al., 2016). Together, these findings suggest that onset of asymptomatic hyperuricemia and gout is more common in summer and autumn, thus new prescriptions for ULDs are also more common during those seasons.

Age and sex are known to be strongly associated with hyperuricemia and gout (Hakoda and Kasagi, 2019; Singh and Gaffo, 2020; Zitt et al., 2020). Consistent with those reports, the proportion of ULDs prescribed in the present study was higher for middle-aged and older as compared with younger patients, and for males as compared with females (Figure 4). However, no significant effect of age or sex was observed in regard to the seasonality of ULD prescription proportions (Figure 3), suggesting some common factors causing seasonal differences independent of age and sex. Differences in ambient temperature and alcohol consumption may account for the higher proportion of new ULD prescriptions for asymptomatic hyperuricemia and gout in summer and autumn as compared to winter. A higher ambient temperature increases the risk of dehydration, which is known to be a factor associated with increased serum urate level through increased purine catabolism and decreased urinary excretion of uric acid (Yamamoto et al., 2004b; Kakutani-Hatayama et al., 2017; Kuge et al., 2017). Consistent with those reports, ambient temperature has been found to be positively correlated with frequency of gout attacks (Neogi et al., 2014). On the other hand, high consumption of alcoholic beverages has been reported to be associated with development of hyperuricemia and gout (Choi et al., 2004a; Wang et al., 2013; Makinouchi et al., 2016; Teramura et al., 2023). Alcoholic beverage consumption is known to increase the level of serum urate due to uric acid production from adenine nucleotide breakdown and decreased uric acid excretion through lactic acid production, in addition to intake of purines contained in those beverages (Yamamoto et al., 2004a; Ka et al., 2005; Yamamoto et al., 2005). Notably, among alcoholic beverages, consumption of beer has been shown to be strongly associated with increased serum urate level and gout (Choi et al., 2004a; Choi and Curhan, 2004; Singh et al., 2011; Zykova et al., 2015), and also to have a correlation with ambient temperature because of higher levels of consumption in summer (Silm and Ahas, 2005; Kaminski et al., 2021). Thus, higher ambient temperature and increased alcohol consumption, especially beer, in summer and autumn as compared to winter may increase the incidence of asymptomatic hyperuricemia and gout in summer and autumn, resulting in new ULD prescriptions more commonly seen in those seasons.

Following stratification by asymptomatic hyperuricemia and gout, new ULD prescriptions for asymptomatic hyperuricemia were significantly lower and for gout significantly higher in spring as compared to winter, whereas those in summer and autumn were similar for both groups (Figure 3). Some previously reported smaller studies noted that onset of gout is more common in the spring (Schlesinger et al., 1998; Choi et al., 2020), though, to the best of our knowledge, there have been no reports indicating that serum urate levels are higher or lower in spring. In Japan, many workplace transfers of patients as well as doctors occur in April, during which a treating physician may refrain from prescribing new ULDs for asymptomatic hyperuricemia patients without symptoms. On the other hand, gout is a painful disease and new ULDs for gout may be more commonly prescribed regardless of workplace transfer issues. Thus, the presence or absence of symptoms may be an important issue affecting the discrepancy regarding new ULD prescriptions in spring between asymptomatic hyperuricemia and gout. Alternatively, factors that cause gout but not hyperuricemia may be present during that season. Additional studies regarding prescription patterns, as well as development of hyperuricemia and gout in the spring will be needed.

As for the significant seasonality regarding new ULD prescriptions shown in subgroup analyses by age and sex, that was also noted with medical institution stratification (Figure 3). The present results suggest that because Japan has a universal health insurance system that allows patients to visit any medical institution and receive medical treatments without restriction, new-onset patients with asymptomatic hyperuricemia or gout were seen regardless of the medical institution, resulting in similar seasonality among the institutions. On the other hand, the Japanese Guidelines for Management of Hyperuricemia and Gout, 3^rd^ edition, recommend administration of ULDs not only to patients with gout but also those with asymptomatic hyperuricemia complicated by renal disease, though not to patients affected by hypertension or heart failure (Hisatome I et al., 2020), in contrast to Western guidelines, such as the 2020 American College of Rheumatology Guideline for the Management of Gout, which does not recommend UDL administration for patients with asymptomatic hyperuricemia (FitzGerald et al., 2020). Further studies are needed to determine the impact of these guidelines on pharmacological management including seasonality of ULD prescriptions for patients with asymptomatic hyperuricemia and gout as a part of clinical practice.

The present study has several important limitations. First, the commercially available JMDC data used in the present study allows extraction of only numbers of patients by age, sex, ICD-10 diagnostic code, and prescription details, thus other information for individual cases could not be obtained. As result, adjustment of results of the present study by use of confounding factors, including age, sex, disease type, coexisting conditions, concomitant medications, and medical institutions, could not be performed. In addition, information related to time-variable characteristics such as coexisting conditions in each patient could not be obtained. Consequently, the study was limited to subgroup analyses based on time-invariant characteristics. Second, the JMDC data used do not include information for individuals aged 75 and older, since those are automatically enrolled in a medical program for elderly and data regarding new ULD prescriptions for such patients were not available. Third, factors not examined included serum urate level, ambient temperature, and alcohol intake, as well as data for degree of obesity, physical activity level, and dietary intake of meat, seafood, fructose, and dairy products, known to be associated with serum urate level and gout (Takahashi et al., 1997; Yamamoto et al., 1997; Choi et al., 2004b; Kaya et al., 2006; Kurajoh et al., 2011; Kurajoh et al., 2020). Fourth, though Japan extends for a long distance from north to south and weather patterns vary considerably in different regions, seasons are classified according to the Japan Meteorological Agency classification and the same throughout the country. Since we were not able to extract region-specific data from the commercially available JMDC data for the present study, analyses that take into account monthly average temperature by region were not performed. Fifth, the number of female patients newly prescribed ULDs was noticeably lower than that of male patients, possibly because new prescriptions for ULDs for treating asymptomatic hyperuricemia and gout are less common in female cases. Inequalities in enrolment of women in clinical trials of ULDs have been reported (Fogacci et al., 2021), which is an issue that should be considered in future studies related to prescription of such drugs. Finally, we defined patients with a code M10 and with/without a code E79.0 as affected by gout, while those with a code E79.0 and without a code M10 were defined as affected by asymptomatic hyperuricemia. However, the distinction between asymptomatic hyperuricemia and gout based on ICD-10 codes may differ from the actual situations, because of the possibility of incorrect entry of the codes or other such errors. Large-scale and more detailed investigations will be necessary to better elucidate the reasons for seasonality of new ULD prescriptions. Nevertheless, the present results obtained by examination of a large dataset of more than 200,000 patients indicate that new prescriptions for ULDs for asymptomatic hyperuricemia and gout are seasonal, and significantly more frequent in summer and autumn.

In conclusion, the present results showed new ULD prescriptions for asymptomatic hyperuricemia and gout were significantly greater in summer and autumn, with no notable difference after stratification by disease type, drug type, and medical institution, or subgrouping by age and sex. These findings suggest that because of seasonal variations in occurrence of asymptomatic hyperuricemia and gout, for which ULDs are routinely prescribed, new ULD prescriptions are more common in summer and autumn in Japan. On the other hand, new ULD prescriptions for asymptomatic hyperuricemia were significantly lower and those for gout significantly higher in spring than winter. It will be necessary to conduct further studies to clarify prescription patterns, as well as factors related to development of hyperuricemia and gout in the spring.

## Data Availability

The raw data supporting the conclusion of this article will be made available by the authors, without undue reservation.
